# Vision driven trailer loading for autonomous surface vehicles in dynamic environments

**DOI:** 10.3389/frobt.2025.1607676

**Published:** 2025-09-22

**Authors:** Jianwen Li, Jalil Chavez-Galaviz, Nina Mahmoudian

**Affiliations:** School of Mechanical Engineering, Purdue University, West Lafayette, IN, United States

**Keywords:** autonomous surface vehicle (ASV), autonomous trailer loading, vision-based navigation, finite state machine (FSM), object detection

## Abstract

Automated docking technologies for marine vessels have advanced significantly, yet trailer loading, a critical and routine task for autonomous surface vehicles (ASVs), remains largely underexplored. This paper presents a novel, vision-based framework for autonomous trailer loading that operates without GPS, making it adaptable to dynamic and unstructured environments. The proposed method integrates real-time computer vision with a finite state machine (FSM) control strategy to detect, approach, and align the ASV with the trailer using visual cues such as LED panels and bunk boards. A realistic simulation environment, modeled after real-world conditions and incorporating wave disturbances, was developed to validate the approach and is available^1^. Experimental results using the WAM-V 16 ASV in Gazebo demonstrated a 100% success rate under calm to medium wave disturbances and a 90% success rate under high wave conditions. These findings highlight the robustness and adaptability of the vision-driven system, offering a promising solution for fully autonomous trailer loading in GPS-denied scenarios.

## 1 Introduction

Autonomous surface vehicles (ASVs) are typically launched and recovered using a crane or a trailer, with human operators overseeing the process. While trailer loading may seem straightforward, it is a technically challenging task that requires precise alignment between the ASV and the trailer, often performed by the operator from a constrained or limited point of view, such as the shore. This task demands skilled maneuvering, particularly in the presence of environmental disturbances like wind and waves. This paper examines the complexities of automating the trailer loading process, focusing on the key challenges in achieving reliable and efficient autonomous docking.

One of the major challenges in ASV docking lies in managing complex problems simultaneously, such as trajectory planning, environmental disturbances, and control constraints. Docking requires precise maneuvering in confined spaces, often near static obstacles, making collision-free trajectory planning a critical component Bitar et al. (2020). However, due to the underactuated nature of most ASVs, their ability to correct lateral errors (sway direction) is limited, necessitating anticipatory alignment to avoid significant corrections near the docking zone. Some of the work of ASV docking balances between minimizing energy consumption and time constraints, as optimizing for one can negatively impact the other Djouani and Hamam (1995). External disturbances, such as waves and currents, further complicate docking by introducing dynamic perturbations, affecting both perception and control and demanding robust feedback mechanisms to maintain trajectory accuracy Martinsen et al. (2019). Traditional PID-based controllers struggle with such dynamic conditions, requiring model predictive control (MPC) or other optimal control strategies to dynamically adjust the ASV’s motion in real-time Martinsen et al. (2019). Despite advancements in trajectory optimization and dynamic positioning, achieving fully autonomous, reliable, and efficient docking remains challenging due to the need for real-time adaptability and robustness against environmental uncertainties.

Automated trailer loading can be seen as a special case of automated docking of autonomous surface vehicles, where an ASV is maneuvered onto a mobile trailer platform. In this study, the platform is carried by a pickup truck, adding unique challenges not present in conventional docking scenarios. A map-based or purely GPS-based approach, such as those used in Bitar et al. (2020) and Martinsen et al. (2019), is insufficient for this application, as the position of the trailer can vary with each attempt due to manual operation of the pickup. Additionally, unlike berthing, which typically occurs in deeper waters, trailer docking happens near the shore, increasing the risk of collisions and potential damage to the propellers of the ASV. These constraints require a precise and adaptive docking strategy.

In particular, some advancements aim to enhance robustness, precision, and operational safety using vision-based and sensor-integrated positioning systems in ASV docking. A vision-based docking approach integrating a virtual force-based strategy and target segmentation has shown promise in coordinated ASV docking with underwater vehicles Dunbabin et al. (2008). The authors of Pereira et al. (2021) propose a volumetric convolutional neural network (vCNN) for detecting docking structures from 3D data, achieving over 96% accuracy in commercial harbors through synthetic datasets combining LiDAR, stereo, GPS, and IMU data. Complementary to this, fiducial marker-based strategies enable ASVs to augment GNSS-RTK and INS systems, providing accurate positioning even in GNSS-compromised areas Digerud et al. (2022). For enhanced redundancy and reliability, a visual-inertial navigation system fuses camera-tag pose estimates with inertial data using an error-state Kalman filter, achieving robust state estimation under urban and challenging weather conditions Volden et al. (2023). Collectively, these innovations provide a foundation for more robust detection and localization of the dock in automated docking tasks.

For Trajectory planning and control, advanced algorithms based on imitation learning Wang et al. (2024), reinforcement learning Lambert et al. (2021); Li et al. (2023), and model-based control Chavez-Galaviz et al. (2024); Li et al. (2024) have been developed for mobile robots in marine and riverine environments. Specifically for the ASV docking, Ahmed and Hasegawa (2013); Im and Nguyen (2018) proposed an artificial neural network (ANN) as a function approximator for the policy, learning to imitate pre-recorded docking demonstrations, and hence learning how to perform the docking maneuvers. In Wang and Luo (2021); Holen et al. (2022); Pereira and Pinto (2024), the ASV docking task was modeled as Markov decision process (MDP) and a deep reinforcement learning agent was trained to perform the docking of an ASV by interacting with the environment. Optimization-based planning Djouani and Hamam (1995); Mizuno et al. (2015); Martinsen et al. (2019); Li et al. (2020) also achieves promising results in ASV docking. These methods allow for explicitly including dynamics and constraints when planning a trajectory using convex optimization.

Despite the growing body of literature on automated docking, studies specifically focused on docking and loading onto trailers remain scarce. The author Abughaida et al. (2024) defined the problem of autonomous trailer loading and explored GPS and AprilTag-based localization, Dubin’s path planning, and model-based trajectory optimization to address autonomous trailer loading tasks. Experiments conducted using a commercial pontoon boat validate the framework’s effectiveness, achieving an 80% overall success rate despite challenges from localization errors and wind disturbances. The method proposed in this work focuses on the perception side of trailer loading without using the GPS or AprilTag, making it more low-cost and easy to adapt to different ASVs and trailers.

The contributions of this paper are threefold. First, we propose a vision-based pipeline for trailer localization, which includes trailer identification, approaching, and loading. Unlike GPS-based methods, this approach does not require prior knowledge of the trailer’s position, making it more adaptable to real-world scenarios where the trailer’s placement may vary. Second, we develop a finite state machine (FSM) control strategy inspired by human experience, where transitions are triggered by the information acquired through the perception system rather than absolute positioning. This strategy accounts for vehicle actuation limitations, practical constraints, failures, retries, and environmental conditions, ensuring robust docking performance. Finally, we introduce an open-source trailer loading simulation environment to validate the proposed automated trailer loading algorithm without relying on GPS, demonstrating its effectiveness under wave disturbances and varying ASV initial placements.

The remainder of this paper is organized as follows: Section 2 formalizes the problem setting in this study. Section 3 details the methodology and system architecture. Section 4 presents the validation results. Finally, conclusions and future work are discussed in Section 5.

## 2 Background

### 2.1 ASV dynamics

A 6-DOF mathematical model of an ASV, is given in Equations 1, 2, can be presented as described in Fossen (2011) when considering that gravity and buoyancy generate restoring forces that cancel out the pitch, roll, and heave motions.

$$\dot{\eta }=J\left(\eta \right)\nu$$ (1)

$$M\dot{\nu }+C\left(\nu \right)\nu +D\left(\nu \right)\nu =\tau +\tau_{\mathrm{waves}}$$ (2)

where $\eta ={\left[x,y,z,\phi ,\theta ,\psi \right]}^{T}$ defines the ASV’s position and orientation in an inertial coordinate system. The speed vector in the body-fixed frame $\nu ={\left[u,v,w,p,q,r\right]}^{T}$ consists of the linear velocities (u,v,w) in the surge, sway, and heave directions, and p,q,r is the rotation velocity. The thrust vector τ contains the force and moment produced by the port and starboard trolling motor commands $a=\left[a_{p},a_{s}\right]$ , respectively. The disturbance $\tau_{w}$ is produced by the wave. The matrix J(η) includes the rotation matrix and transformation matrix and is used as a transform from the body-fixed frame to the earth (inertial) fixed frame. M , C(ν) , and D(ν) represent the inertia matrix, the Coriolis and centripetal force matrix, and the damping matrix, respectively.

### 2.2 Problem definition

The problem of trailer loading for ASVs involves developing a system that enables the ASVs to autonomously and accurately dock onto a trailer in dynamic and uncertain environments. The trailer is fixed in position and orientation at $\eta_{t}$ . The ASV must detect and localize the trailer’s position using onboard sensors. The objective is to generate control commands that minimize the error between ASV’s η and the trailer’s $\eta_{t}$ while avoiding collision. The trailer loading is successful if errors in the x-axis, y-axis, and yaw are smaller than the terminal error thresholds $\epsilon_{\mathrm{long}}$ , $\epsilon_{\mathrm{lat}}$ , and $\epsilon_{\mathrm{ang}}$ . Loading is considered a failure if the ASV fails to reach the trailer loading zone within the termination time $T_{\max}$ .

### 2.3 Digital twin

**FIGURE 1:**
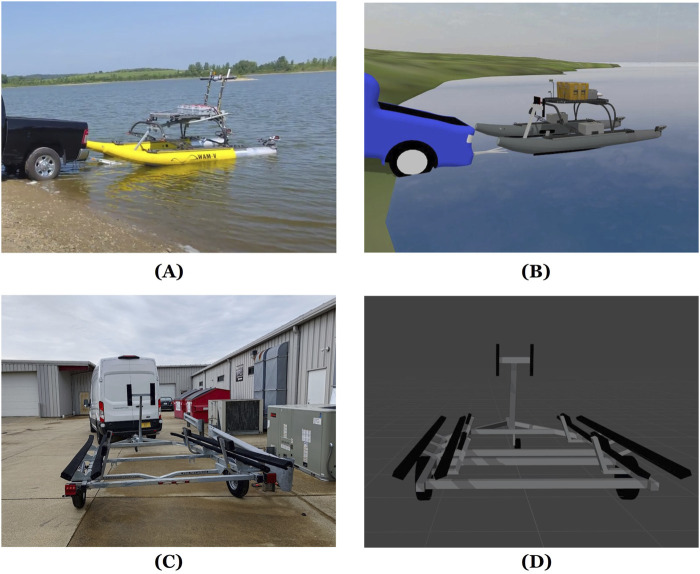
Illustrations of the trailer loading setup and simulation environment: **(A)** Real-world trailer loading with the ASV, **(B)** Gazebo-based simulation environment, **(C)** Physical trailer hardware, and **(D)** Blender trailer model.

A simulation environment for the trailer loading scenario has been created, as shown in Figure 1. A trailer and a pickup truck model are created in Autodesk Inventor. A field test environment is also modeled to mimic Lake Harner, IN, using Blender. The dimensions of the boat and trailer have been measured accurately, ensuring that the spatial relationships in the simulation match those in reality. This is important for docking and loading scenarios, where even small errors in dimensions could affect the system’s performance. The trailer loading environment is built based on the Virtual RobotX (VRX) simulator Bingham et al. (2019). Plugins from VRX are used to calculate the wave forces, and Open Dynamics Engine (ODE) Smith (2005) provides the simulation of the rigid-body dynamics.

The sensor noise of IMU has been measured from real-world tests and injected into the simulation, making the simulation more realistic. The noise profile included Gaussian-distributed random errors with standard deviations matching the real-world IMU data. Camera noise was also modeled by injecting per-pixel Gaussian noise into the image stream, with zero mean and a standard deviation of 0.007, approximating the visual noise observed in real camera feeds. The motor delay was modeled using the default settings in the VRX simulator, which treats the motor as a second-order system. This approach captures the dynamic response of the motor, including its rise time and settling time.

The simulation is also able to generate wind and wave disturbances. Since wind disturbance mainly affects the control accuracy and rejecting wind disturbance has been widely studied Pereira et al. (2008); Chavez-Galaviz et al. (2023); Abughaida et al. (2024), this study focuses exclusively on wave disturbances, which pose greater challenges to vision-drive system during trailer loading. The simulation uses the Pierson-Moskowitz wave spectra Fréchot (2006) to generate realistic wave patterns. The spectrum $S_{B}\left(\omega \right)$ in Equation 3 captures the mean energy in a wave field as a function of angular frequency ω and is specified by the peak frequency $\omega_{p}$ and an independent significant wave height ${\bar{H}}_{s}$

$$S_{B}\left(\omega \right)=\frac{1.25}{4}\left(\frac{\omega_{p}^{4}}{\omega^{5}}\right){\left({\bar{H}}_{s}\right)}^{2}\exp\left[-\frac{5}{4}{\left(\frac{\omega_{p}}{\omega }\right)}^{4}\right]$$ (3)

A user-specified non-dimensional gain value $K_{H}=\frac{{\bar{H}}_{s}}{H_{s}}$ is defined as the ratio of the desired significant wave height to the significant wave height. In this work, we validate the trailer loading system’s performance using different gain values from 0 to 0.5 to generate different wave disturbances.

## 3 Methodology

**FIGURE 2:**
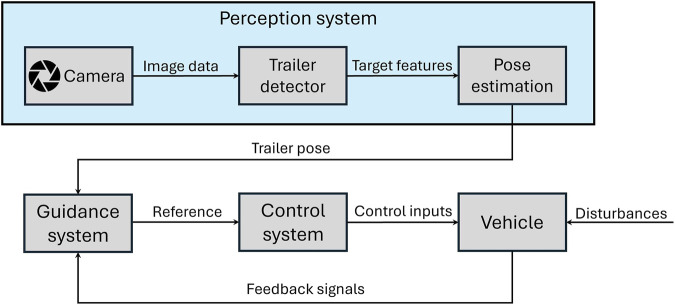
Overview of the proposed vision-based trailer loading system. The architecture integrates real-time image processing and a finite state machine (FSM)-based control strategy to enable robust alignment and docking in dynamic environments.

In this section, we present the system architecture and methodology for autonomous trailer loading as depicted in Figure 2, focusing on the integration of image processing and control strategies. The primary challenge lies in accurately detecting and localizing the trailer’s features (e.g., the LED panel and bunk boards) in real-time, despite varying environmental conditions such as wave disturbances. Our approach leverages a flat finite state machine (FSM) to guide a modular control structure, ensuring precise alignment and loading of the boat onto the trailer. The following subsections detail the image processing pipeline for feature detection and the control strategy for error correction.

### 3.1 Perception system

**FIGURE 3:**
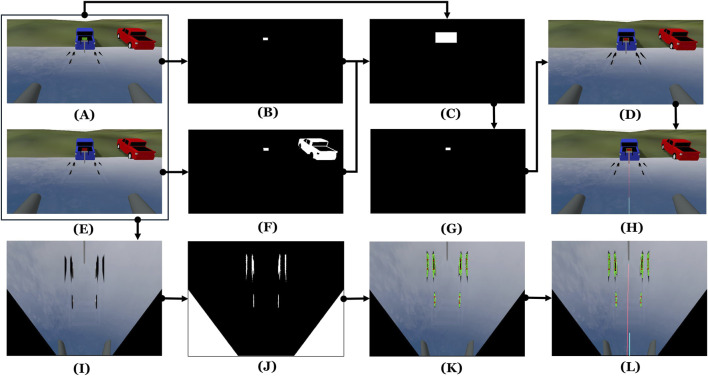
Image processing pipeline for the perception system: **(A–B)** Panel detection sequence when the panel is green; **(C)** region of interest (ROI); **(D)** Detected panel; **(E–F)** detection sequence when the panel is red; **(G)** mask to detect green or red color within the ROI, **(H)** detected panel with a line to the robot to calculate heading error, **(I)** bird’s-eye view (BEV) transformation of the scene; **(J–L)** detection of bunk boards and centerline extraction for lateral error estimation.

The image processing sequence of the perception system is illustrated in Figure 3. One front-facing camera is used to detect the features of the trailer for autonomous loading. We mainly focus on two features: an LED panel and black horizontal bunk boards. The LED panel is 350 by 200 mm. It is mounted on the trailer, displaying a sequence of colors with a specific timing. There are 8 bunk boards in total, 4 long ones to support the hull of the vehicle and 4 short ones to support the bow of the vehicle.

While Olson (2011) are a reliable and widely used visual fiducial system for precise object detection and localization, An LED panel might be more suitable for this scenario due to the following reasons: LED panels emit light, making them highly visible even in low-light or challenging environments, where AprilTags may be hard to detect without sufficient external illumination. LED panels can display time-based color patterns (e.g., red for 2 s followed by green for 1 s in a repeating cycle), providing an additional layer of information for dynamic localization, an advantage not available with static fiducial markers like AprilTags. LED panels can be detected with lower-resolution cameras due to their brightness, while AprilTags often require higher-resolution cameras for reliable recognition, especially at long distances.

The detection of the target LED panel involves capturing video frames and processing them in real-time. The panel displays a repeating color sequence: red for $T_{\mathrm{red}}$ seconds, followed by green for $T_{\mathrm{red}}$ seconds, and repeating. The total cycle time is denoted as $T_{\mathrm{cycle}}$ . Each frame $I_{\mathrm{RGB}}$ is converted to HSV color space as $I_{\mathrm{HSV}}$ for effective color segmentation. Binary masks $M_{\mathrm{red}}$ and $M_{\mathrm{green}}$ , given by Equations 4, 5 are then applied to isolate red and green regions based on predefined HSV thresholds $\theta_{\mathrm{red}}$ and $\theta_{\mathrm{green}}$ , respectively.

$$M_{\text{red }}\left(x,y\right)=\left\{\begin{array}{cc} 1\; & \text{ if }I_{HSV}\left(x,y\right)\in \theta_{\text{red }},\\ 0\; & \text{ otherwise } \end{array}\right.$$ (4)

$$M_{\text{green }}\left(x,y\right)=\left\{\begin{array}{cc} 1\; & \text{ if }I_{HSV}\left(x,y\right)\in \theta_{\text{green }},\\ 0\; & \text{ otherwise } \end{array}\right.$$ (5)

The contours of these regions are extracted, and their centers are tracked to distinguish the target panels. The panel candidate is defined as a tuple $P_{i}=\left(x_{i},y_{i},w_{i},h_{i},t_{i},col_{i}\right)$ . For each candidate, the center, shape, detection timestamps, and corresponding colors are recorded, retaining only recent k detections within the defined timing window. The candidate set is denoted as $P=\left\{P_{i-k+1},P_{i-k+2},\ldots ,P_{i}\right\}$ .

A pattern-matching algorithm is then applied to verify whether the panel follows the red-to-green timing sequence. Let $P_{\text{red}}$ and $P_{\text{green}}$ denote the subsets of P corresponding to red and green detections, respectively. The first detected candidates in the red and green subsets are defined in Equation 6.

$$P_{1}^{\text{red}}=\underset{P_{i}\in P_{\text{red}}}{\mathrm{arg min}}t_{i},\;P_{1}^{\text{green}}=\underset{P_{i}\in P_{\text{green}}}{\mathrm{arg min}}t_{i}$$ (6)

As the first detected candidates in the red and green subsets, respectively. A set P is considered valid $P_{\text{valid}}$ if it meets the condition from Equation 7:

$$\left|t_{1}^{\text{red}}-t_{1}^{\text{green}}\right|\geq \min\left(T_{\text{green}},T_{\text{red}}\right)$$ (7)

Then, the target panel is identified by Equation 8 as the latest candidate in $P_{\text{valid}}$ :

$$P_{\text{target }}=\underset{P_{i}\in P_{\text{valid}}\left(t\right)}{\mathrm{arg max}}t_{i}.$$ (8)

The position of the identified panel $\left(x_{\mathrm{target}},y_{\mathrm{target}}\right)$ is returned as the center of the region of interest (ROI). We assume the ROI is a rectangle and its shape is decided by the height and width of the panel $\left(h_{\mathrm{target}},w_{\mathrm{target}}\right)$ . To account for the movement of the vehicle, the ROI needs to be larger than the target panel. Let α=2 be the scaling factor. The shape of the ROI becomes $\left(h_{\mathrm{ROI}},w_{\mathrm{ROI}}\right)=\left(\alpha \cdot h_{\mathrm{target}},\alpha \cdot w_{\mathrm{target}}\right)$ . This approach ensures accurate detection of the target panel in scenarios involving multiple segments of similar color, size, and shape.

Using the ROI as a mask, we effectively filter out objects with colors similar to those of the LED panel, focusing exclusively on potential target panels within the defined area. By applying color segmentation and contour detection within the ROI, we accurately identify the LED panel and determine its center point as $\left(x_{\mathrm{panel}},y_{\mathrm{panel}}\right)$ . The camera is at $\left(x_{\mathrm{camera}},y_{\mathrm{camera}}\right)$ . The angular offset between the camera’s optical axis and the panel’s center is then calculated, providing the angular error denoted as $e_{\mathrm{ang}}$ from Equation 9:

$$e_{\mathrm{ang}}=\arctan\left(\frac{x_{\mathrm{panel}}-x_{\mathrm{camera}}}{y_{\mathrm{panel}}-y_{\mathrm{camera}}}\right)$$ (9)

This angular error is critical for aligning the camera or robot’s orientation with the detected panel. Additionally, the position and shape of the panel, determined from its bounding contour, are used to dynamically refine and update the ROI in subsequent frames. This adaptive ROI ensures that the system maintains focus on the correct panel while excluding irrelevant objects, even in complex environments with multiple similarly colored or shaped elements.

The bird’s-eye view (BEV) transformation is essential for simplifying spatial understanding and enabling accurate distance measurements. By removing perspective distortion, BEV allows objects to appear at their true scale and relative positions, making it easier to measure distances and plan motions. This is particularly useful for tasks such as collision avoidance and alignment with the trailer.

**FIGURE 4:**
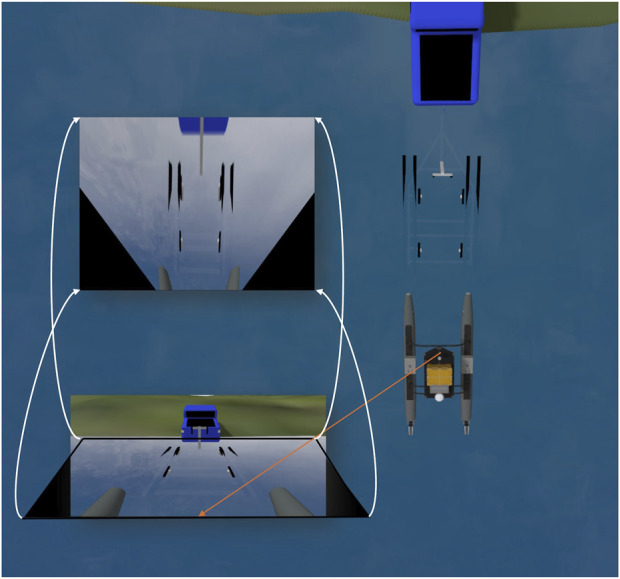
Bird’s-eye view (BEV) transformation process. A homography matrix is used to convert the camera image to a top-down view, enabling more accurate spatial measurements for alignment and motion planning.

Figure 4 depicts the process of BEV transformation. Once the raw RGB image $I_{\mathrm{RGB}}$ is captured from the camera, it is transformed into the BEV. Equation 10 according to Szeliski (2022), is used to transform a point (x,y) in the image plane to a point $\left(x^{\prime },y^{\prime }\right)$ in the bird’s-eye view plane is:

$$\left[\begin{array}{c} x^{\prime }\\ y^{\prime }\\ w \end{array}\right]=H\left[\begin{array}{c} x\\ y\\ 1 \end{array}\right]$$ (10)

Where H is the homography matrix and w is the scale factor used for normalization. To get the normalized BEV coordinates $\left(x_{\mathrm{BEV}},y_{\mathrm{BEV}}\right)$ , divide the point $\left(x^{\prime },y^{\prime }\right)$ in the bird’s-eye view plane by w : $x_{\mathrm{BEV}}=\frac{x^{\prime }}{w},y_{\mathrm{BEV}}=\frac{y^{\prime }}{w}$ .

Once the conversion is complete, the position of each pixel in the image becomes the same as the actual position of the feature with respect to the real-world camera frame. In this way, BEV removes perspective distortion, making objects appear at their true scale and relative positions. This representation makes it easier to measure distances and relative orientations between objects, thus simplifying collision avoidance and motion planning by reducing the complexity of interpreting depth and perspective.

Once a BEV image $I_{\mathrm{BEV}}$ is generated, the black bunk boards are extracted by masking defined in Equation 11 with the threshold $\theta_{\text{bunk}}$ and blob detection.

$$M_{\text{bunk }}\left(x,y\right)=\left\{\begin{array}{cc} 1\; & \text{ if }I_{\mathrm{BEV}}\left(x,y\right)\in \theta_{\text{bunk }},\\ 0\; & \text{ otherwise } \end{array}\right.$$ (11)

The bunk boards are classified into left and right groups based on their centroids and slopes. The center line of bunk boards is calculated as y=m⋅x+c . We calculate the distance from the camera to the center line as the lateral error, which is described by Equation 12:

$$e_{\mathrm{lat}}=\frac{m\cdot x_{\mathrm{camera}}-y_{\mathrm{camera}}+c}{\sqrt{m^{2}+1}}$$ (12)

The longitudinal error $e_{\mathrm{long}}$ is determined by interpolating from a pre-constructed table as shown in Table 1 that relates pixel counts from the LED panel $c_{\mathrm{panel}}$ to corresponding $e_{\mathrm{long}}$ values. This table contains pairs of pixel counts and the associated longitudinal errors $e_{\mathrm{long}}$ , representing the relationship between the area of the LED panel and the actual longitudinal error. When a pixel count is observed from the LED panel, the closest values in the table are identified, and linear interpolation is used to estimate the corresponding $e_{\mathrm{long}}$ . The interpolation process is given by Equation 13:

$$e_{\mathrm{long}}=\text{Interpolate}\left(c_{\mathrm{panel}}\right)$$ (13)

**TABLE 1:** Table of pixel count and longitudinal error pairs for interpolation.

LED panel pixel count $c_{\mathrm{panel}}$	Longitudinal error $e_{\mathrm{long}}$ (m)
72	20.0
84	15.0
264	10.0
793	5.0
1739	3.0
2,475	2.0
8,345	1.0
32,189	0.5
42,012	0.3
56,552	0.1

Where $c_{\mathrm{panel}}$ is the observed pixel count from the LED pthe image plane to a panel, and $e_{\mathrm{long}}$ is the interpolated longitudinal error.

### 3.2 Control strategy

**FIGURE 5:**
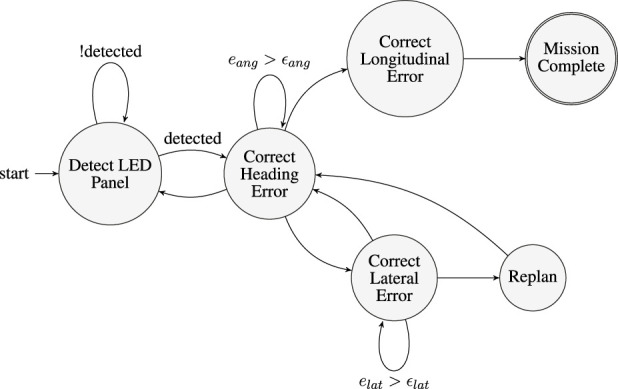
Finite State Machine (FSM) for autonomous trailer docking which is designed based on operator experience. The FSM consists of 6 high-level states. Transitions between states are driven by real-time perception feedback and threshold-based error conditions. The FSM ensures robust error correction, failure recovery, and mission completion.

We chose a hierarchical control structure to control the ASV to load on the trailer. The hierarchical control structure includes a finite state machine (FSM), as shown in Figure 5, and low-level controllers for each state.

The FSM control strategy is designed by incorporating operator experience and encoding intuitive methods for docking and navigation. It is capable of handling unexpected disruptions during the loading process. The FSM defines six high-level states and four state variables. The states are: Detect LED Panel (initial state), Correct Heading Error, Correct Lateral Error, Correct Longitudinal Error, Replan, and Mission Complete.

The four state variables are: (1) LED panel detected, (2) lateral error below threshold $e_{\mathrm{lat}}$ , (3) heading error below threshold $e_{\mathrm{ang}}$ , and (4) longitudinal error below threshold $E_{x}$ . Transitions between states are governed by the values of these variables. A dedicated low-level controller is implemented for each state to carry out the required maneuvers.

In the starting state, it will rotate itself until it detects the LED panel. Once the LED panel is detected, it will enter the Correct Heading Error state. A PI controller is utilized to minimize the heading error $e_{\mathrm{ang}}$ so that the ASV can align with the trailer. The PI controller for heading error correction was tuned to achieve a balance between responsiveness and stability. The proportional gain was set to ensure rapid convergence, while the integral gain was chosen to minimize steady-state error. This combination allows the ASV to align with the trailer efficiently. After the $e_{\mathrm{ang}}$ is smaller than the threshold $\epsilon_{\mathrm{ang}}=0.05rad$ , the system will first try to minimize the lateral error $e_{\mathrm{lat}}$ , and then minimize the longitudinal error $e_{\mathrm{long}}$ . In the Correct Lateral Error state, a time-based controller is used to decrease $e_{\mathrm{lat}}$ . The ASV will turn clockwise if $e_{\mathrm{lat}}>0$ or counterclockwise if $e_{\mathrm{lat}}<0$ for $\left|k_{1}e_{\mathrm{lat}}\right|$ steps with constant angular velocity $r_{1}$ , then move forward for $\left|k_{2}e_{\mathrm{lat}}\right|$ steps with a constant linear velocity $u_{1}$ where $k_{1}$ and $k_{2}$ are tunable parameters to make the time-based controller and minimize the $e_{\mathrm{lat}}$ efficiently. However, when misalignment happens due to sudden wave of perception error, if $e_{\mathrm{lat}}$ is larger than the threshold $\epsilon_{\mathrm{ang}}=0.1\;m$ and the boat is closer than 0.5m to the bunk boards, the system will enter the Replan state, in which the boat moves backwards with a constant linear velocity $u_{2}$ until it has enough space to realign itself with the target so that the boat can keep correcting the error while avoiding collision to the trailer. Once sufficient space is achieved, the system re-enters the Correct Heading Error state to correct its position and resume the docking process. If the ASV briefly loses visual contact with the LED panel, it stops its motion and transitions to the Detect LED Panel state.

Finally, if both the $e_{\mathrm{lat}}$ and $e_{\mathrm{ang}}$ are smaller than the thresholds, the boat will enter the moving forward state. In the Correct Longitudinal Error state, the boat uses another PI controller to decrease the $e_{\mathrm{long}}$ . If $e_{\mathrm{long}}$ is smaller than the threshold $\epsilon_{\mathrm{long}}=0.1\;m$ , we consider the loading to be successful and stop the motors.

## 4 Results

This section examines the impact of wave disturbances on the behavior of the autonomous surface vehicle (ASV) and evaluates the performance of the autonomous trailer loading system under varying environmental conditions. The experiments were designed to assess how the ASV’s trajectory and replanning behavior adapt to increasing wave disturbances and how these adaptations affect overall docking success, task completion time, and operational efficiency. All simulations were conducted on a desktop computer equipped with a GeForce RTX 2080 GPU and an Intel Core i7-8700 CPU. By systematically varying the wave disturbance gain $K_{H}$ and analyzing the ASV’s responses, the results provide insights into the robustness and adaptability of the control and replanning strategies for safe and reliable autonomous docking operations.

### 4.1 Impact of wave

**FIGURE 6:**
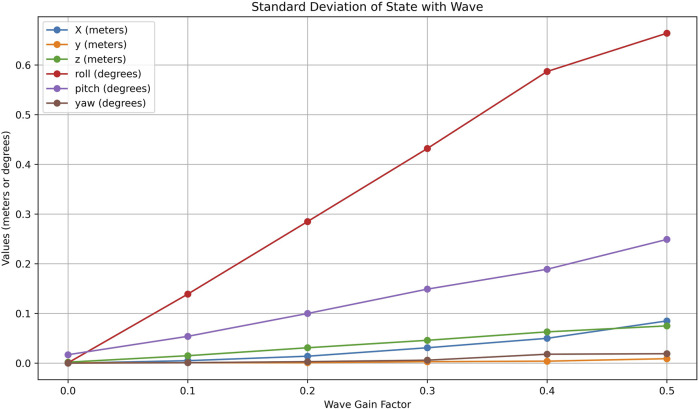
Effects of varying wave disturbance gains on ASV state variables. Plots show standard deviation in position and orientation, revealing increased roll and heave under higher wave amplitudes, which impact perception and stability.

The result in Figure 6 reveals the impact of wave amplitude on the system’s displacement and rotational dynamics. Starting with linear displacement, the displacement on the x-axis shows a clear linear increase in the amplitude of the wave, highlighting that forward movement is significantly influenced by wave intensity. Similarly, the displacement on the z-axis exhibits a noticeable upward trend, indicating that the system experiences a greater heave motion as wave amplitudes grow. In contrast, the displacement on the y-axis remains nearly constant across all wave values, suggesting that the lateral displacement is minimally affected and the system maintains stability in the lateral direction.

For rotational dynamics, the roll angle shows the most significant variation, increasing substantially with wave amplitude. This indicates that the system undergoes considerable tilting about the x-axis as the waves intensify, likely caused by uneven wave forces acting on the structure. The pitch also increases steadily, though at a lower rate than the roll, reflecting the forward-backward tilting caused by the waves. The yaw angle, on the other hand, shows only minimal variation, suggesting that the system experiences very slight rotational motion about the z-axis, with minor asymmetries in wave interaction causing this effect.

These observations have important physical implications, particularly for systems such as maritime vehicles. The changes in z-axis displacement, roll, and pitch with wave amplitude suggest potential stability issues under rough sea conditions, with the system becoming more prone to tilting and heaving, which can significantly affect the ASV’s perception systems, especially cameras. These rotations may introduce noise and misalignment in sensor readings, reducing the accuracy of object detection, localization, and tracking.

**FIGURE 7:**
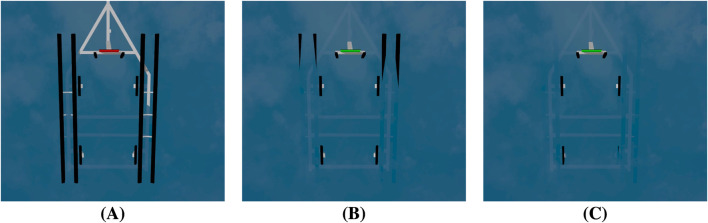
Visualization of trailer submersion under increasing wave heights. **(A)** Low waves: bunk boards remain fully exposed. **(B)** Moderate waves: partial submersion of long bunk boards. **(C)** High waves: complete submersion of long and side bunk boards, reducing visibility for detection.

Additionally, the varying wave heights in Figure 7 will significantly affect image processing for autonomous trailer loading. As the waves rise, the trailer and its bunks become increasingly submerged. The submerged bunks make it difficult for the image processing system to accurately detect the trailer’s position and orientation. Therefore, autonomous trailer loading systems will need to employ robust image processing techniques that can handle these challenges.

### 4.2 Trailer loading

**FIGURE 8:**
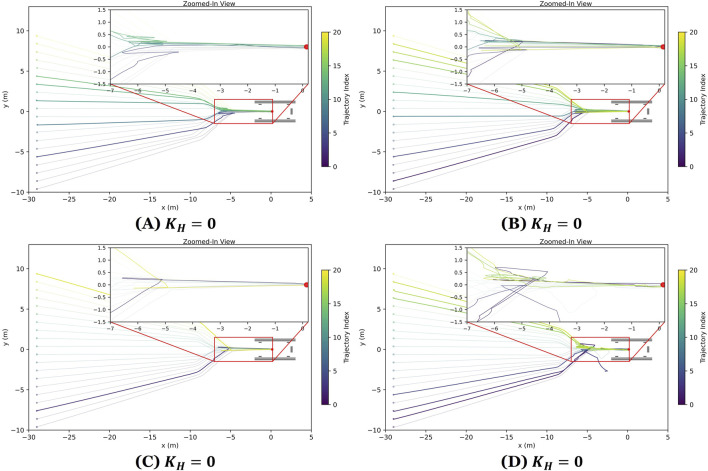
ASV docking trajectories under varying wave disturbance levels including **(A)** calm wave disturbances $\left(K_{H}=0.0\right)$ , **(B)** mild wave disturbances $\left(K_{H}=0.1\right)$ , **(C)** moderate wave disturbances $\left(K_{H}=0.3\right)$ , and **(D)** high wave disturbances $\left(K_{H}=0.5\right)$ . The initial positions are distributed 30m longitudinally and span and ±9.5 m laterally from the trailer. Red rectangle highlights the docking zone, and the zoomed-in view emphasizes the final approach and docking accuracy near the trailer. The highlighted trajectories correspond to trials with replanning or failures.

To evaluate the performance of the proposed autonomous trailer loading system under varying environmental conditions, a total of 80 experiments were carried out. Each trail corresponds to one of four wave disturbance gain levels $K_{H}:0.0,0.1,0.3,0.5$ , with 20 tests per condition. The termination time $T_{\max}$ was set to 100s . In all tests, the wave’s direction was aligned with the AVS’s x-axis. The ASV was initialized at a distance of 30 m from the trailer, with a fixed longitudinal offset of 29m and a lateral distance variation ranging from −9.5 m and +9.5 m . The resulting docking trajectories are illustrated in Figure 8, and performance metrics are summarized in Table 2.

**TABLE 2:** Performance of the autonomous trailer loading system under varying wave disturbance gain levels, highlighting the system’s robustness across increasing environmental disturbances. Metrics include docking success rate, average completion time ( ± standard deviation), number of replanning events, cumulative time spent replanning, and number of failed docking attempts. Results are based on 20 trials per condition.

Wave gain	0	0.1	0.3	0.5
Success Rate(%)	100	100	100	90
Avg. Completion Time (s)	609.1±79	591.2±99.1	624.3±91.7	728.5±168.2
Replan Events (#)	2	7	5	6
Avg. Replan time (s)	7.3	28.5	23.0	34.1
Failures (#)	0	0	0	2

The docking system achieved a 100% success rate in calm, mild, and moderate wave conditions ( $K_{H}$ levels 0.0, 0.1, and 0.3, respectively). Under the high wave disturbance condition $\left(K_{H}=0.5\right)$ , the success rate dropped slightly to 90% , with two docking failures recorded.

Average task completion time ranged from (591.2±99.1s) to (728.5±168.2 s) . The shortest times were observed at $K_{H}=0.1$ , while the longest occurred at $K_{H}=0.5$ —reflecting the additional complexity in maintaining alignment under severe wave motion. Replanning events were triggered when the ASV deviated significantly from its intended trajectory due to perception errors or environmental forces. As expected, both the frequency and the duration of replanning increased with higher wave gain. For example, the number of replan events increased from 2 to 6, with the average replanning time increasing from 7.3 to 34.1 s between calm $\left(K_{H}=0.0\right)$ and severe $\left(K_{H}=0.5\right)$ scenarios. Throughout these trials, the system operated in real time with an image processing rate of 30 FPS. The mean image process time was 0.0078 s, with a standard deviation of 0.0015 s, indicating the system’s capability to process sensor data and make decisions in real-time.

To better understand how wave disturbances affect system performance, we compared success rates, completion times, and the frequency of replanning events across different wave gain levels. The data revealed clear trends: as wave intensity increased, task completion times generally rose, with the most noticeable difference occurring between the low disturbance case $\left(K_{H}=0.1\right)$ and the high disturbance case $\left(K_{H}=0.5\right)$ . While the system maintained high success rates of 90% or greater across all scenarios, performance declined under the most challenging conditions. At $K_{H}=0.5$ , the system experienced a 10% failure rate and a 23% increase in average completion time compared to calm conditions. Additionally, the number of replanning events increased alongside wave intensity, suggesting that replanning plays a key role in maintaining robust docking performance in dynamic environments.

**FIGURE 9:**
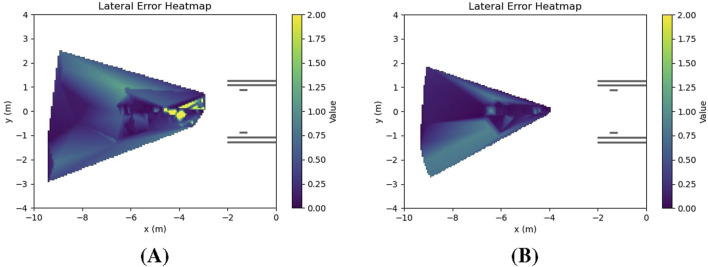
Heatmaps comparing lateral error accuracy **(A)** Calm disturbances $\left(K_{H}=0\right)$ ; **(B)** High wave disturbances $\left(K_{H}=0.5\right)$ . The heatmaps illustrate the spatial distribution of the accuracy of lateral error measurements. Brighter regions indicating higher errors between the perceived and actual lateral position of the trailer.

**FIGURE 10:**
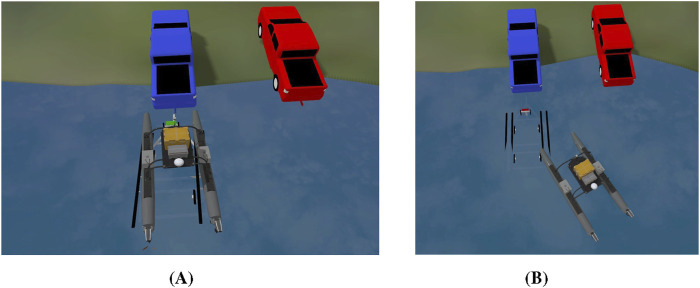
Examples of ASV docking failures under high wave disturbances. **(A)** ASV reaches the trailer but is misaligned; **(B)** ASV fails to load onto the trailer. Failures are caused by significant perception errors and large pose deviations.

Figure 9 shows heat maps of the lateral error accuracy in scenarios with calm wave disturbance and high wave disturbance. As wave intensity increased, the perception error grew - especially in estimating lateral alignment - lowering the accuracy of the lateral error measurements and contributing directly to the two observed failures. Figure 10 illustrates these failure scenarios. The first failure case is shown in 10 (A). The ASV successfully docked but was misaligned. Due to friction between the hull and the trailer bunks, it was unable to correct its heading and realign after docking, resulting in a misaligned final position. For the second failure case in Figure 10B, the ASV failed to board the trailer entirely. After initially correcting the erroneous lateral error estimate, it encountered the short bunk supporting the bow of the vehicle, causing it to get stuck before completing the docking process. These failures highlight the critical impact of wave-induced perception errors on docking performance.

**FIGURE 11:**
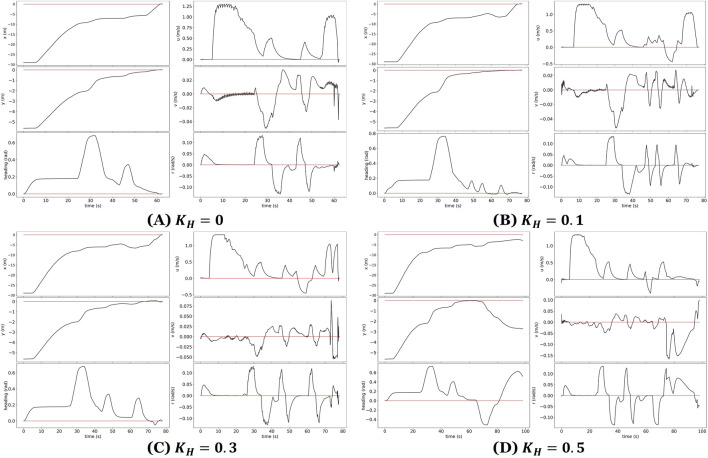
State plots showing ASV pose and control responses during trailer loading under varying wave conditions. **(A–C)** Successful trials with increasing wave intensity; **(D)** failed trial under large disturbances. Red lines indicate target values; black lines represent ASV state trajectories.

Despite these challenges, the FSM-based control system successfully handled most alignment errors, demonstrating robustness in the presence of moderate to severe environmental disturbances. Figure 11 presents state trajectories for four representative trials. These plots provide valuable insight into the dynamic behavior of an ASV during autonomous loading onto a trailer under varying wave disturbance conditions. The plots show increased oscillations in the ASV’s pose and control signals as wave intensity rises. While the control system remained stable in mild and moderate scenarios, large disturbances introduced substantial deviations that exceeded the system’s ability to compensate.

To contextualize these results, we also qualitatively compared our system to the existing GPS- and AprilTag-based trailer loading framework by Abughaida et al. (2024). That method achieved an 80% success rate in physical experiments but relied on GNSS infrastructure and fiducial markers. In contrast, our vision-only approach reached a 100% success rate in mild and moderate conditions and maintained a 90% success rate under high wave disturbances—all without GPS or external markers. These results highlight the adaptability, cost-effectiveness, and infrastructure independence of our method, making it highly suitable for real-world, unstructured environments.

## 5 Conclusion and future work

This paper presents a novel vision-based framework for automating trailer loading of autonomous surface vehicles (ASVs) in GPS-denied environments. The proposed system leverages a camera-based perception pipeline and a finite state machine (FSM) paired with PID controllers to achieve precise alignment and docking. Evaluated in a high-fidelity simulation environment with realistic disturbances and sensor noise, the framework achieved a high success rate of 97.5% , demonstrating strong robustness against wave-induced challenges.

While the system performed reliably in most scenarios, occasional failures under high wave disturbances were observed due to occlusion or submersion of trailer features, which affected perception accuracy. These were partially mitigated by the FSM’s replan mechanism, though reliance solely on vision introduces limitations in extreme conditions. Future work will explore sensor fusion and adaptive control strategies to further enhance robustness.

The modular architecture of the framework enables scalability to different ASV sizes and trailer designs. The LED-based localization system provides a consistent visual reference, while the FSM and PID control components can be retuned to account for changes in dynamics. Although the current system assumes full LED panel visibility, planned extensions include adding redundancy, fallback strategies, and real-world testing. In addition, the system’s lightweight computational requirements make it well-suited for deployment on embedded platforms, reducing dependence on high-performance computing hardware and enhancing its practicality for real-world ASV applications.

However, several limitations remain. Larger ASVs typically exhibit different inertial and hydrodynamic properties, requiring more precise system identification and control retuning. The risk of LED marker occlusion increases with vessel size or environmental complexity, which may necessitate redundancy in marker placement or sensor fusion with alternative modalities. Furthermore, the current system relies on black lumber as reference landmarks, which are difficult to detect under low-light conditions, limiting nighttime operation. Addressing these challenges is crucial for enabling robust, scalable deployment in diverse real-world environments.

Overall, this work contributes a practical, extensible solution to autonomous trailer docking, laying a strong foundation for more intelligent, robust, and scalable maritime autonomy systems.

## Data Availability

The raw data supporting the conclusions of this article will be made available by the authors, without undue reservation.
